# Two Varieties of Intrahepatic Cholangiocarcinoma

**DOI:** 10.1155/1994/43495

**Published:** 1994

**Authors:** David M. Nagorney

**Affiliations:** Mayo Clinic 200 First Street SW Rochester MN 55905 USA


cheap. Whether initial treatment should be extended to long-term regimes is a point of contention beyond the scope of the present article.

An extremely important point emphasised by the authors is the definition of treatment failure, particularly with reference to injection sclerotherapy. They have clearly shown that repeated attempts to manage bleeding by sclerotherapy are associated with diminishing returns and it is suggested that 2 attempts at endoscopic treatment is all that is justified before recourse to other measures. The results of stapling transection observed in the present trial would strongly support this technique for the early rescue of patients who have failed endoscopic therapy. Any such intervention should be considered over a period of hours rather than days after the time of presentation. The only reservation with respect to this advice may be the possible adverse effect of staple transection upon any subsequently planned liver transplantation.

### REFERENCES

1. Huizinga, W., Angorn, P. and Baker, L. (1985) Esophageal transection versus injection sclerotherapy in the management of bleeding esophageal varices in patients at high risk. *Surg. Gynecol Obstet.*, **160**, 539–546
2. Teres, J., Bordas, J.M., Bravo, D. *et al.* (1987) Sclerotherapy vs distal splenorenal shunt in the elective treatment of variceal hemorrhage: A randomized controlled trial. *Hepatology*, **7**, 430–436
3. Paquet, K-J, Feussner, H. (1985) Endoscopic sclerosis and esophageal balloon tamponade in acute hemorrhage from esophagogastric varices: A prospective controlled randomized trial. *Hepatology*, **5**, 580--583
4. Moreto, M., Zaballa, M., Bernal, A. *et al.* (1988) A randomized trial of tamponade or sclerotherapy as immediate treatment for bleeding esophageal varices. *Surg. Gynecol. Obstet.*, **167**, 331--334
5. Westaby, D., Hayes, P., Gimson, A. *et al.* (1989) Controlled clinical trial of injection sclerotherapy for active variceal bleeding. *Hepatology*, **9**, 274–277

Dr D. Westaby
Consultant Physician and Gastroenterologist
Charing Cross Hospital
Fulham Palace Road
London W6 8RF
United Kingdom

### TWO VARIETIES OF INTRAHEPATIC CHOLANGIOCARCINOMA

### ABSTRACT

*Yamamoto, J., Kosuge, T., Takayama, T., Shimada, K., Makuuchi, M., Yoshida, J., Sakamoto, M., Hirohashi, S., Yamasaki, S. and Hasegawa, H. (1992) Surgical treatment of intrahepatic cholangiocarcinoma: Four patients surviving more than five years. Surgery; 111: 617–622*

**Background. To find the rational surgical strategy for the treatment of intrahepatic cholangiocarcinoma (ICC), clinical features of ICC were studied in 20 patients who underwent hepatic resection in the National Cancer Center Hospital from 1980 to 1990.**

**Methods. According to the morphologic pattern, we classified the ICCs into two subcategories, mass-forming and infiltrating, which correlated with their biologic behavior.**

**Results. Of 10 patients who underwent hepatectomy for mass-forming ICC, three survived more than 5 years without recurrence. The 1-, 3-, and 5-year survival rates were 59.3%, 44.4%, and 44.4%, respectively. Of 10 patients who underwent hepatectomy for infiltrating ICC, one survived more than 5 years without recurrence. The 1-, 3-, and 5-year survival rates were 72.0%, 27.0%, and 27.0%, respectively. The pathologic findings and recurrences indicated that the salient feature of the mass-forming type was its tendency for intrahepatic metastasis especially near a main lesion, and of the infiltrating type was the infiltrative spread via Glisson's capsule and hilar lymph nodal metastasis.**

**Conclusions. An anatomic and extensive liver resection should be performed for mass-forming ICC, whereas a hepatectomy with excision of the extrahepatic bile duct and hilar lymph nodal dissection is recommended for infiltrating ICC. (Surgery 1992; 111: 617–22)**

**From Department of Surgery, National Cancer Center Hospital, and Pathology Division, National Cancer Center Research Institute, Tokyo, Japan, and First Department of Surgery, Shinshu University School of Medicine, Matsumoto, Japan.**

### PAPER DISCUSSION

KEY WORDS: Intrahepatic cholangiocarcinoma, hepatic resection

Over the last decade the clinicopathology of intrahepatic cholangiocarcinoma (ICC) has been elucidated more clearly. ICC, the second most frequent histotype of primary hepatic malignancy, has proven itself formidable. Yamamoto and colleagues have emphasized this fact in their detailed report of only four patients with ICC surviving five years or more after resection. Their message is both encouraging and sobering.

ICC or peripheral cholangiocarcinoma accounts for 10–20% of primary hepatic malignancies[2,3]. In contrast to hepatocellular carcinoma, concurrent chronic liver disease exists less frequently[3]. The clinical presentation of ICC is not pathognomonic. Gross morphology of ICC is similar to that of hepatocellular carcinoma and is equally divided between massive and infiltrating types. Although Yamamoto *et al.* reported only their experience with patients who underwent resection, other investigators have shown infrequent resectability because of the advanced diseasestage at diagnosis[2–4]. Similar detailed pathologic studies of ICC have shown that intrahepatic tumor spread and regional lymph node and distant organ metastases occur in nearly 70% and 85% of patients respectively[4,5]. Yamamoto *et al.* also found that intrahepatic vascular invasion, intrahepatic metastases, bile duct invasion and lymph node metastases occurred in nearly 50% of their patients. These pathologic findings affirm the acknowledged aggressive biologic behavior of ICC. Not surprisingly resectability is infrequent. Even when ICC is resectable, lobar or extended lobar resections are generally required to encompass all gross disease. Despite extensive hepatic resections reported by Yamamoto *et al.* herein and others,[2-4] five-year disease-free survival is rare. The only inexplicable exception to all other reports is that by Iwatsuki and Starzl[6] who found that ICC was not associated with a poor prognosis, though actual five-year survival was estimated. No report to date has addressed the relationship of TNM stage and survival for this histotype.

What is the clinical impact of the report on ICC by Yamamoto *et al.*? First, their report establishes that surgical therapy has a potentially curative role for this primary hepatic malignancy, albeit infrequently. Clearly surgical therapy should not be abandoned based on the histology of preoperative tumor biopsy alone. Moreover, careful staging evaluation is required to define resectability because of the high risk of metastatic disease with this histotype. Secondly, subsequent reports of the outcome of surgically treated patients with ICC are warranted to determine factors predictive of both resectability and survival in patients with ICC. Reports on ICC are few. Indeed, surgeons are just beginning to learn of this tumor. Additional information regarding staging, resectability rates, and outcomes will aid in the development of future treatment strategies for patients with ICC. Finally, as with other malignancies associated with a poor prognosis and requiring major procedures with significant operative risk, surgeons need to critically define the selection criteria for those patients likely to benefit from resection. Fully 50% of the patients reported by Yamamoto *et al.* survived one year or less. One wonders whether resection had any impact on disease progression in these patients. Clinical performance status of patients and assessment of the quality of life both before and after surgery must be addressed in the future to justify the effort required in the management of patients with ICC.

In summary, Yamamoto *et al.* have called further attention to the challenge of ICC. Although they have provided a glimmer of hope for both patients and surgeons alike by documenting five year survival in four patients, they have fully recognized limitations in resection alone. Perhaps the stimulus of this report will spurn others to critically review their experiences and uncover other salient features of ICC which will lead to improved outcome from surgical management.

### REFERENCES

1. Yamamoto, J., Kosuge, T., Takayama, T., Shimada, K., Makuuchi, M., Yoshida, J., Sakamoto, M., Hirohashi, S., Yamasaki, S., Hasegawa, H. (1992) Surgical treatment of intrahepatic cholangiocarcinoma: Four patients surviving more than five years. *Surgery*, **111**, 617–622
2. Kawarada, Y., and Mizumota, R. (1984) Cholangiocellular carcinoma of the liver. *Am. J. Surg.*, **147**, 354–359
3. Schlinkert, R.T., Nagorney, D.M., van Heerden, J.A., Adson, M.A. (1992) Intrahepatic cholangiocarcinoma: Clinical aspects, pathologyand treatment. *HPB Surg.*, **5**, 95–101
4. Kawarada, Y., and Mizumoto, R. (1990) Diagnosis and treatment of cholangiocellularcarcinoma of theliver.*Hepatogastroenterol*, **37**, 176–181
5. Nakajimi, T., Kondo, Y., Miyazaki, M., Okui, K. (1988) A histopathologic study of 102 cases of intrahepaticcholangiocarcinoma: Histologic classification and modes of spreading. *Human Pathology*, **19**, 1228–1234
6. Iwatsuki, S., and Starzl, T.E. (1989) Experience with resection of primary hepatic malignancy. *Surg. Clin. North Am.*, **69**, 315--322

David M. Nagorney, M.D.
Mayo Clinic
200 First Street SW
Rochester, MN 55905

